# High-Resolution Tracking of Aging-Related Small Molecules: Bridging Pollutant Exposure, Brain Aging Mechanisms, and Detection Innovations

**DOI:** 10.3390/bios15040242

**Published:** 2025-04-11

**Authors:** Keying Yu, Sirui Yang, Hongxu Song, Zhou Sun, Kaichao Wang, Yuqi Zhu, Chengkai Yang, Rongzhang Hao, Yuanyuan Cao

**Affiliations:** 1 Department of Toxicology and Sanitary Chemistry, School of Public Health, Capital Medical University, Beijing 100069, China; yukeying@mail.ccmu.edu.cn (K.Y.); 2418037@mail.ccmu.edu.cn (K.W.); 2 Beijing Key Laboratory of Environment and Aging, Capital Medical University, Beijing 100069, China; 3 School of Basic Medical Sciences, Capital Medical University, Beijing 100069, China; sryoung2023@mail.ccmu.edu.cn (S.Y.); vae514songhx@mail.ccmu.edu.cn (H.S.); sunzhou@mail.ccmu.edu.cn (Z.S.); moosnow@mail.ccmu.edu.cn (Y.Z.); 4 Beijing Friendship Hospital, Capital Medical University, Beijing 100069, China; caneyang@mail.ccmu.edu.cn

**Keywords:** brain aging, environmental pollutants, bioactive small molecules, electrochemical sensor

## Abstract

Brain aging is a complex process regulated by genetic, environmental, and metabolic factors, and increasing evidence suggests that environmental pollutants can significantly accelerate this process by interfering with oxidative stress, neuroinflammation, and mitochondrial function-related signaling pathways. Traditional studies have focused on the direct damage of pollutants on macromolecules (e.g., proteins, DNA), while the central role of senescence-associated small molecules (e.g., ROS, PGE2, lactate) in early regulatory mechanisms has been long neglected. In this study, we innovatively proposed a cascade framework of “small molecule metabolic imbalance-signaling pathway dysregulation-macromolecule collapse”, which reveals that pollutants exacerbate the dynamics of brain aging through activation of NLRP3 inflammatory vesicles and inhibition of HIF-1α. Meanwhile, to address the technical bottleneck of small molecule spatiotemporal dynamics monitoring, this paper systematically reviews the cutting-edge detection tools such as electrochemical sensors, genetically encoded fluorescent probes and antioxidant quantum dots (AQDs). Among them, AQDs show unique advantages in real-time monitoring of ROS fluctuations and intervention of oxidative damage by virtue of their ultra-high specific surface area, controllable surface modification, and free radical scavenging ability. By integrating multimodal detection techniques and mechanism studies, this work provides a new perspective for analyzing pollutant-induced brain aging and lays a methodological foundation for early intervention strategies based on small molecule metabolic networks.

## 1. Introduction

Aging is an inescapable natural law in the course of biological life. Studies have found a significant positive correlation between aging and increased incidence of metabolic-associated diseases such as cardiovascular disease and Alzheimer’s disease [1,2]. The brain, as one of the most important organs in the human body, is greatly affected in the aging process. Recent epidemiological studies have demonstrated that the exposure to environmental pollutants accelerates brain aging, leading to cognitive decline and memory impairment [3,4], and may also exacerbate the progression of neurodegenerative diseases [5,6]. Recent studies have shown that environmental stresses (e.g., nanoparticles in air pollutants) may accelerate brain aging by inducing oxidative stress and inflammatory responses. It is worth noting that the high carbon emissions of conventional building materials [7] may indirectly exacerbate such environmental risks, while the promotion of bio-based materials may reduce this potential threat. However, the specific molecular mechanisms have not been fully elucidated, and the related signaling pathways and pathological regulatory networks still need to be further revealed.

Previous research has primarily focused on the damage caused by pollutants to macromolecules such as proteins and DNA, while overlooking the role of aging-related small molecules as upstream regulators in the brain aging signaling pathway during pollutant-induced brain aging processes [8,9]. Currently, the pathological mechanisms by which environmental pollutants accelerate brain aging by inducing oxidative stress [10], neuroinflammation [11], and mitochondrial dysfunction [12] have now been widely demonstrated. Based on their core biological functions, this review classifies aging-related bioactive small molecules into the following three categories: oxidative stress-related molecules (e.g., reactive oxygen species (ROS), reactive nitrogen species), neuroinflammation-related molecules (e.g., prostaglandin E2, glutamate), and mitochondrial damage biomarkers (e.g., lactate, pyruvate). Since aging-related bioactive small molecules play an important role in regulating cellular signaling networks [13], precise monitoring of their content becomes a critical and indispensable aspect.

Methods commonly used to detect small molecules include chromatography, mass spectrometry, fluorescence imaging, and electrochemical methods. However, traditional chromatography and mass spectrometry techniques are difficult to realize real-time monitoring of small molecule metabolic systems under pollutant exposure due to insufficient spatial and temporal resolution, leading to long-term reliance on static and fragmented data for mechanism studies [14]. In recent years, breakthroughs in electrochemical sensing, fluorescence imaging, and other technologies have provided new tools for real-time dynamic monitoring [15,16]. The technological innovations have provided key theoretical tools for precise intervention in brain aging caused by environmental pollutants.

This review focuses on the role of aging-related small molecules in brain aging mechanism under environmental pollutants. We systematically summarize breakthroughs in small molecule dynamic regulation models and detection technologies and innovatively propose a cascade effect framework of “small molecule metabolism imbalance—signaling pathway dysregulation—macromolecule dysfunction”. This framework provides new perspectives on the understanding of pollutant-induced brain aging. At the same time, we explored early intervention strategies and novel detection technologies based on small molecule metabolism to provide theoretical basis and technical support for the precise prevention and control of brain aging.

## 2. Possible Mechanisms by Which Environmental Pollutants Accelerate Brain Aging Through Bioactive Small Molecules

### 2.1. Oxidative Stress Signaling Pathway

#### 2.1.1. The Enhancement of Oxidative Stress During Brain Aging

**Figure 1:**
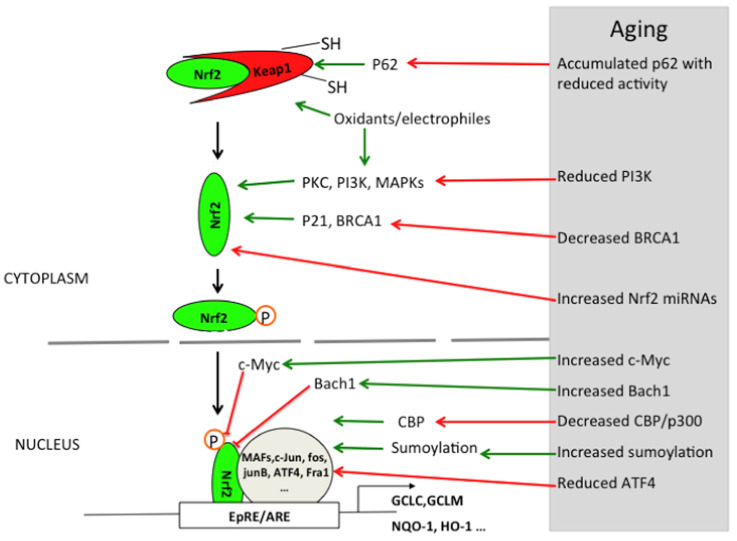
Pattern diagram of age-related changes in Nrf2 regulatory system during brain aging process. Reprinted with permission from Ref. [19]. Copyright 2015 Elsevier.

The brain contains a high content of unsaturated fatty acids that are prone to peroxidation, and it requires a very large amount of oxygen per unit weight (accounting for approximately 20% of the total amount used by humans) [17]. Therefore, the brain is highly susceptible to oxidative stress. With aging, the α subunit of mitochondrial F1 ATP synthase decreases, leading to a reduction in ATP production and an increase in ROS generation [18]. However, during the aging process, the responsiveness of oxidative stress-related signaling pathways such as Nrf2/ARE to oxidative stress significantly declines. This decline is manifested not only in the baseline level of Nrf2 but also in its ability to be induced by oxidative stress (Figure 1) [19]. The intracellular antioxidant defense system gradually weakens, and the activities of antioxidant enzymes such as glutathione peroxidase (GPx) and catalase decrease, leading to a decline in the ability to scavenge ROS [20]. ROS attack the unsaturated fatty acids in the cell membrane, triggering lipid peroxidation reactions and generating lipid peroxidation products such as malondialdehyde and 4-hydroxy-2-nonenal (4-HNE) [21,22]. These products further damage the structure and function of the cell membrane, reducing its fluidity and affecting the functions of membrane proteins such as ion channels and transporters. This membrane damage further impacts the cell’s signal transduction and metabolic functions, exacerbating the cell’s oxidative stress state. This imbalance in oxidative stress not only directly damages cellular components through pathways such as lipid peroxidation but also triggers a series of consequences such as inflammation and apoptosis through multiple signaling pathways [22], thus leading to various aging-related chronic diseases [23]. However, apart from the aging process itself, environmental pollutants may also exacerbate this oxidative stress process. Next, we will explore how environmental pollutants accelerate the process of brain aging by regulating oxidative stress-related pathways.

#### 2.1.2. The Mechanisms by Which Environmental Pollutants Exacerbate Oxidative Stress

**Figure 2:**
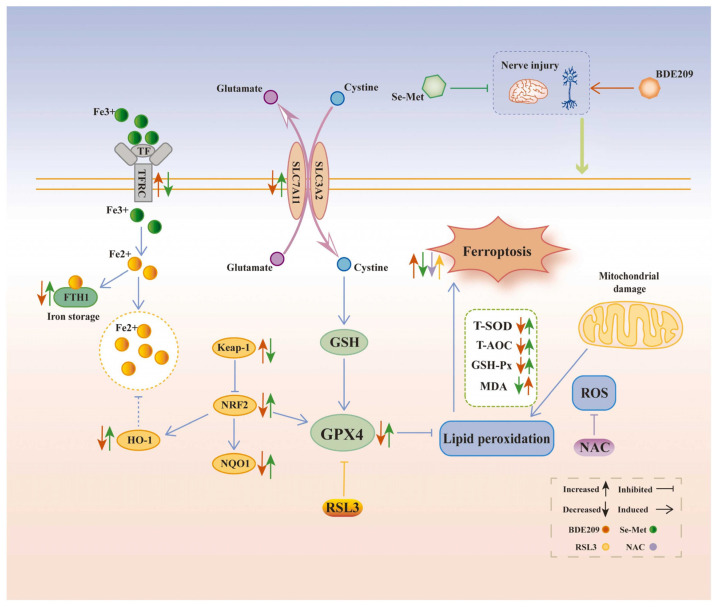
Schematic diagram of the protective effect of methionine selenium in BDE209 induced OS and iron toxicity Reprinted with permission from Ref. [30]. Copyright 2024 Elsevier.

The intracellular antioxidant defense system is mainly regulated by the Nrf2/ARE pathway, and HIF-1α can participate in some antioxidant reactions under hypoxic conditions [24,25]. Nrf2 (nuclear factor E2-related factor 2) is the core transcription factor of the cell’s antioxidant defense system. Under normal physiological conditions, Nrf2 binds to Keap1 (Kelch-like ECH-associated protein 1) and is anchored in the cytoplasm [24]. Keap1 mediates the ubiquitination-dependent degradation of Nrf2 through the Cullin3-dependent E3 ubiquitin-ligase complex, thus inhibiting the activity of Nrf2 [26]. When cells are under oxidative stress, ROS or electrophilic substances modify the key cysteine residues on Keap1, leading to a conformational change in Keap1, which then releases Nrf2. Subsequently, Nrf2 translocates to the nucleus and binds to the antioxidant response element (ARE), activating the expression of a series of antioxidant genes [26,27]. Under the condition of environmental pollutant exposure, the activation pattern of the Nrf2 pathway may change. Through their study, Chu, Chen et al. found that the expression of Nrf2 was significantly increased during long-term exposure to high concentrations of PM_2.5_ in an animal model. This study highlights the direct effect of a single pollutant on the Nrf2 pathway and emphasizes the importance of Nrf2 in cellular antioxidant defense [28]. Additionally, Dong, Bowen et al. deeply explored the effects of single pollutant exposure on brain tissues and their intervention mechanisms and found that pollutant exposure induced oxidative stress and ferroptosis, whereas selenomethionine (Se-Met) could attenuate pollutant-induced oxidative damage and neurotoxicity through activation of the Nrf2/GPX4 pathway [29]. To reveal the complex regulatory mechanisms of the Nrf2 pathway by combined exposure to multiple pollutants, Zhao, Hongjing et al. further investigated the effects of environmentally relevant concentrations of insecticides and antibiotics on brain tissues, which significantly activated the Nrf2 pathway, regardless of whether the exposure was alone or in combination (Figure 2) [10]. This suggests that the Nrf2 pathway can coordinate antioxidant responses to protect cells from oxidative damage in the face of combined exposure to multiple pollutants. Therefore, the Nrf2 pathway plays a crucial protective role in response to oxidative stress induced by single or multiple environmental pollutants, and an in-depth study of its regulatory mechanism is of great significance for the development of environmental toxicology and related protective strategies.

**Figure 3:**
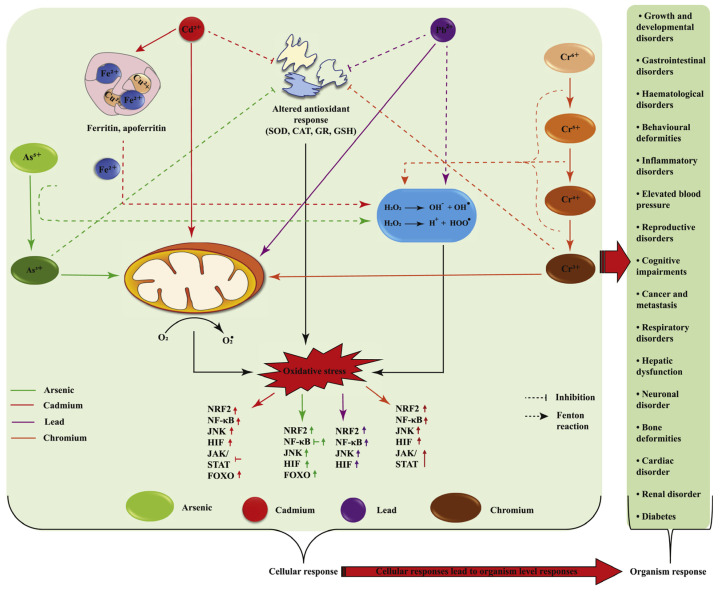
Metal pollutants cause damage to organs in the body through oxidative stress. Reprinted with permission from Ref. [34]. Copyright 2021 Elsevier.

HIF-1α (hypoxia-inducible factor-1α) is another key transcription factor in cells’ response to oxidative stress. Under normal circumstances, hydroxylation modification of HIF-1α enables it to bind to VHL, which is then degraded by the E3 ubiquitin ligase [25,30]. However, under hypoxic or oxidative stress conditions, the hydroxylation modification of HIF-1α is reduced, allowing it to stably exist and translocate to the nucleus [25]. In the nucleus, HIF-1α binds to HIF-1β to form a dimer, activating the expression of various downstream genes, including antioxidant enzymes (such as HO-1, NQO1), energy metabolism-related enzymes (such as LDHA), and angiogenesis factors (such as VEGF). This enhances the cell’s antioxidant capacity and promotes the adaptation of cell energy metabolism and angiogenesis, helping the cell survive under hypoxic and oxidative stress conditions [25,31]. Under normal physiological conditions, the expression level of HIF-1α is tightly regulated, and its stability is mainly affected by the combined action of oxygen concentration, oxidative stress status, and various enzymes (such as prolyl hydroxylase, ubiquitin–proteasome system, etc.). However, when the body is exposed to environmental pollutants, this balance is disrupted, triggering a series of complex biological effects. Chang et al. found that acute methylmercury (MeHg) exposure significantly reduced the protein expression level of HIF-1α and inhibited the expression of several of its downstream target genes in rat brain tissues and primary cultured astrocytes, revealing the inhibitory effect of MeHg on HIF-1α [32]. Unlike MeHg, the mode of regulation of HIF-1α by heavy metals is more complex. In the nervous system, vascular dysfunction is considered to be a key causative factor in neurodegenerative diseases such as Alzheimer’s disease and Parkinson’s disease [33]. Paithankar et al. found that Cr^6+^ exposure sustained activation of the HIF-1α protein and facilitated the expression of VEGF (vascular endothelial growth factor) by inducing ROS generation, thus enhancing abnormal vasculature generation (Figure 3) [34]. This may indirectly exacerbate brain aging and neurodegenerative lesions. This study not only reveals the complex regulation of HIF-1α by heavy metal pollution but also explains the possible role of HIF-1α in the development of neurodegenerative diseases. In addition, Zheng et al.’s study found that PM_2.5_ exposure led to aberrant activation of the HIF-1α signaling pathway and induced ferroptosis in different tissues, ultimately leading to functional damage [35]. This further reveals the multilevel effects of different pollutants on the HIF-1α signaling pathway and deepens our understanding of the mechanisms of environmental pollution-induced neurological injury.

In addition to the Nrf2/ARE and HIF-1α pathways, the intracellular antioxidant defense system is also regulated by other signaling pathways, such as NF-κB, PI3K/Akt, FoxO, and AMPK. These pathways are not only closely related to oxidative stress but also interact with processes such as neuroinflammation, mitochondrial function, and metabolic regulation. In the following chapters, we will further explore the roles of these pathways in pollutant-induced brain aging.

### 2.2. Neuroinflammation Signaling Pathways

#### 2.2.1. The Enhancement of Neuroinflammation During Brain Aging

Under normal physiology, the neuroinflammatory response is tightly regulated. NF-κB, a key transcription factor in the inflammatory response, is normally bound to the inhibitory protein IκB and exists in the cytoplasm in an inactive state [36,37]. Activation and inhibition of NLRP3 inflammatory vesicles are also in dynamic balance [38]. However, with the onset of brain aging, this regulation becomes progressively imbalanced. During brain aging, factors such as oxidative stress and chronic inflammation enhance the activity of the NF-κB signaling pathway, leading to the degradation of IκB, the release of NF-κB and its translocation into the nucleus, which in turn initiates the transcription of inflammation-related genes, resulting in a significant increase in the expression of pro-inflammatory cytokines such as TNF-α and IL-6 [37,39]. Meanwhile, with NLRP3 inflammatory vesicles in the microenvironment of senescent cells, the original inhibitory mechanism is weakened, and the assembly and activation process is more likely to occur. The activation of NLRP3 inflammatory vesicles prompts the activation of caspase-1, which leads to the release of large amounts of inflammatory factors, such as IL-1β and IL-18, and further exacerbates neuroinflammatory responses [40]. Liang R et al. found that the levels of pro-inflammatory markers are higher in aging organisms and that the inflammatory process is closely linked to the activation of NLRP3 inflammatory vesicles [41]. This enhanced neuroinflammatory response not only affects the normal function of nerve cells but also disrupts the homeostasis of the entire nervous system, becoming an important feature of the brain aging process [42].

#### 2.2.2. The Mechanisms by Which Environmental Pollutants Exacerbate Neuroinflammation

**Figure 4:**
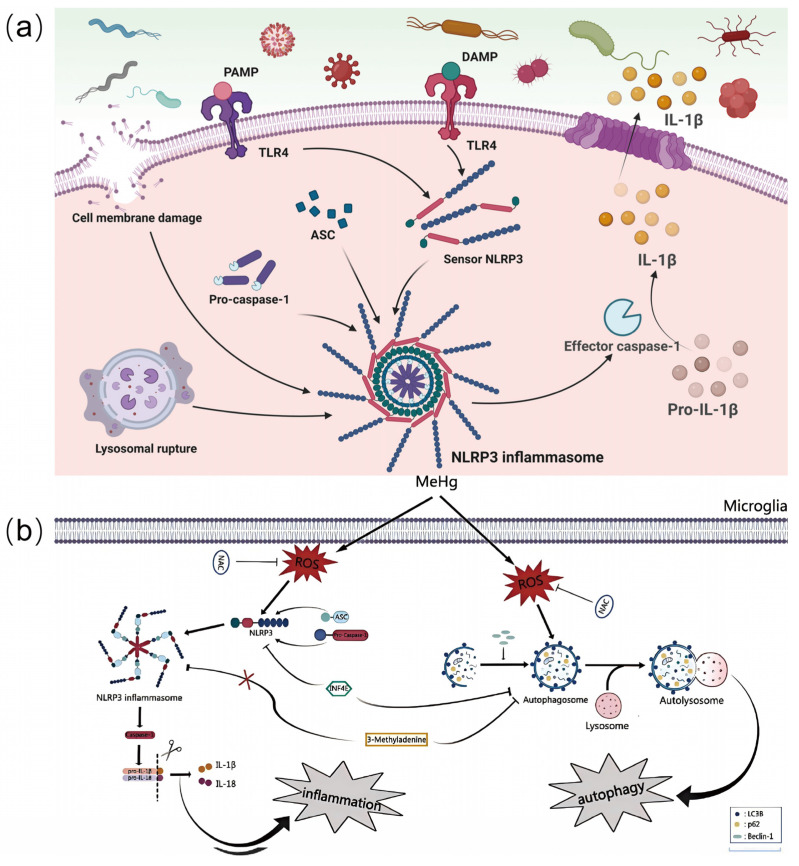
Pollutants affect the body through neuroinflammatory factors. (**a**) Two-step activation of NLRP3 inflammasome at the molecular level. Reprinted with permission from Ref. [41]. Copyright 2024 Springer Nature. (**b**) Schematic diagram of the proposed signaling pathway involving microglia in methylmercury-induced inflammation and autophagy. Reprinted with permission from Ref. [11]. Copyright 2024 Elsevier.

The intracellular neuroinflammatory response is mainly regulated by the NF-κB signaling pathway and the NLRP3 inflammatory vesicle-associated pathway. The proper functioning of the NF-κB signaling pathway relies on stringent and complex mechanisms. When cells are in a resting state, NF-κB is tightly bound to IκB and stably exists in the cytoplasm [36,37]. When cells are stimulated, upstream kinases such as IκB kinase (IKK) are activated [36,43]. The activated IKK phosphorylates and modifies IκB, causing rapid degradation of IκB by the ubiquitin–proteasome system. This allows NF-κB to dissociate from its bound state and translocate into the nucleus [37]. In the nucleus, NF-κB binds to specific κB sites on DNA, initiating a series of transcriptional processes of inflammation-related genes and ultimately producing pro-inflammatory cytokines such as TNF-α and IL-6 [39]. The activation of NLRP3 inflammatory vesicles is similarly complex and delicate. Two key signals are required for its activation: first, an initiating signal, which drives the upregulation of the expression of inflammasome-associated components such as NLRP3, ASC, and pro-caspase-1, followed by a triggering signal, which causes a conformational change in NLRP3 and binds to ASC, which in turn recruits pro-caspase-1 to form a complex that ultimately leads to caspase- 1 activation, cleaving precursors of inflammatory factors such as IL-1β and IL-18, maturing and releasing them (Figure 4a) [40,41].

And environmental pollutants can affect the above pathways through multiple sites of action. Studies have shown that pollutant exposure leads to aberrant protein expression, causing inflammatory responses. Li X et al. found that methylmercury was able to increase the expression of NLRP3, ASC, and Caspase-1 proteins in BV2 cells, inducing the production of ROS and the activation of the NLRP3 inflammatory vesicle (Figure 4b) [11]. Similarly, Wang X et al. found that the expression of inflammation-related proteins such as p-p65, TNF-α, IL-6, and IL-8 was increased and the NF-κB pathway was activated after PM_2.5_ treatment of cells [44]. This process cannot be separated from the effects of pollutants on gene expression, in which epigenetic effects are particularly important. Grieco M et al. found that β-HCH can regulate the NF-κB pathway, induce histone acetylation modifications, and promote inflammatory factor expression [45]. In addition, further exploration of the whole process of pollutants from affecting gene expression to generating inflammatory responses plays an important role in our study of the relationship between pollutants and brain aging. Zhao YS et al. found that lead exposure altered the levels of gene expression-related proteins, which in turn activated NF-κB and increased the release of pro-inflammatory cytokines, through the study of rat cerebral cortex [46]. Various environmental pollutants directly or indirectly affect the pathway mechanisms through the above pathways, exacerbating the imbalanced state of the pathway and inducing excessive neuroinflammatory responses, thus aggravating the process of brain aging.

### 2.3. Mitochondrial Dysfunction Signaling Pathways

#### 2.3.1. The Phenomenon of Mitochondrial Dysfunction During Brain Aging

Mitochondria, as the “energy factories” of cells, play an important role in maintaining the normal function of the nervous system [19]. Under normal physiological conditions, cells recognize healthy and damaged mitochondria through the PINK1/Parkin pathway and selectively degrade damaged mitochondria through autophagy to maintain mitochondrial quality and function [47].

**Figure 5:**
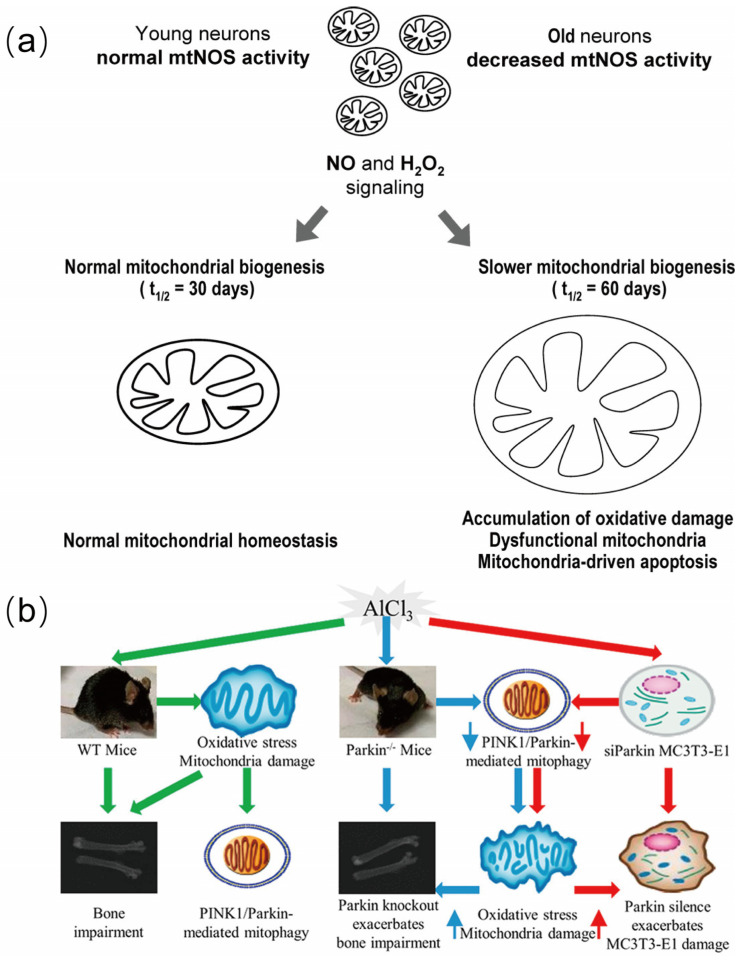
Pollutants destroy the body through mitochondrial autophagy. (**a**) Schematic diagram illustrating the role of mitochondria in biosynthesis under NO and H_2_O_2_ stimulation, as well as the disruption of mitochondrial and cellular homeostasis in the elderly brain. Reprinted with permission from Ref. [50]. Copyright 2008 Wiley-VCH. (**b**) Pollutants are based on the PINK/Parkin pathway, leading to mitochondrial autophagy. Reprinted with permission from Ref. [58]. Copyright 2021 American Chemical Society.

Oxidative phosphorylation (OXPHOS) is the main pathway for ATP production, while antioxidant systems (e.g., SOD2, GPx) tightly regulate ROS levels, which together ensure energy metabolism homeostasis [48]. However, with age, the regulatory mechanisms of the PINK1/Parkin signaling pathway become imbalanced. The mitochondrial autophagy process is either over-activated, resulting in excessive mitochondria being consumed and affecting cellular energy supply; or the function is impaired and unable to remove damaged mitochondria in a timely manner, which allows the damaged mitochondria to accumulate inside the cell, generating a large amount of ROS and further damaging the mitochondria and the neuronal cells [49]. Boveris et al. found that in aged animals, the rate of mitochondrial respiration and electron transfer is reduced, the membrane potential is decreased, phospholipid and protein oxidation products are elevated, and the size and fragility of mitochondria increase (Figure 5a) [50]. At the same time, oxidative phosphorylation processes were severely affected. The activity of the mitochondrial respiratory chain complex is significantly reduced, electron transfer is blocked, and oxidative phosphorylation is uncoupled, leading to reduced ATP production and disturbed cellular energy metabolism [51]. In addition, impaired oxidative phosphorylation processes lead to increased ROS production, which triggers lipid peroxidation and damages the structure and function of neuronal cell membranes and other biological membranes [52]. These are likewise important features of the brain aging process [42].

#### 2.3.2. The Mechanisms by Which Environmental Pollutants Exacerbate Mitochondrial Dysfunction

Dysfunction of mitochondria during brain aging increases the sensitivity of neuronal cells to environmental pollutants [53]. Environmental pollutants can further affect the signaling pathways related to mitochondrial function through multiple pathways, leading to increased mitochondrial dysfunction and thus exacerbating the process of brain aging [54]. And mitochondrial function is mainly related to the PINK1/Parkin pathway and oxidative phosphorylation system. In terms of the PINK1/Parkin signaling pathway, when the mitochondrial membrane potential decreases, PINK1 is stabilized and autophosphorylated at the outer mitochondrial membrane and recruits the cytoplasmic E3 ubiquitin ligase Parkin, which ubiquitinates outer mitochondrial membrane proteins (e.g., VDAC1, MFN2), which are then recognized by the articulin p62/SQSTM1, which recognizes the ubiquitination mark and recruits LC3-II-encapsulated autophagosomes to encapsulate the damaged mitochondria, where the autophagosome fuses with the lysosome and the contents are broken down by hydrolases to recover amino acids and lipids [47,55]. In terms of the oxidative phosphorylation system, it establishes a proton gradient through the electron transport chain (ETC) complex (I–IV), which drives ATP production by ATP synthase (complex V) [52,56]. Through the above pathway mechanisms, normal mitochondrial morphology and function are maintained.

It has been shown that many environmental pollutants exacerbate mitochondrial dysfunction in senescent cells by affecting the above pathway, further exacerbating the process of brain aging. Pollutants can inhibit mitochondrial function by inhibiting normal changes in the mitochondrial electron transport chain. Zolkipli-Cunningham Z et al. found that MPTP and its active metabolites, MPP⁺, fisetinone, and paraquat, interfere with the oxidative phosphorylation process and affect the normal synthesis of ATP [57]. To further explore the cascade of this process leading to normal energy metabolism in mitochondria, Shang Y found that ultrafine particulate carbon black (uBC) causes a loss of mitochondrial membrane potential and a decrease in ATP levels, which leads to mitochondrial damage, apoptosis, and respiratory dysfunction [12]. Mitochondrial dysfunction and structural damage ultimately lead to mitochondrial autophagy and death. Cui Y found that aluminum chloride exposure induces oxidative stress and activates PINK1/Parkin-mediated mitochondrial autophagy (Figure 5b) [58]. The complete process described above due to pollutant exposure further worsens brain aging.

### 2.4. Other Related Mechanisms

#### 2.4.1. Multiple Roles of Nrf2, NF-κB, and NLRP3 Inflammatory Vesicle Signaling Pathways

Nrf2 is not only a central regulator of cellular antioxidant response but also plays an important role in suppressing neuroinflammation. Studies have shown that Nrf2 indirectly inhibits inflammatory responses by upregulating antioxidant genes, such as superoxide dismutase 2 (SOD2), catalase, heme oxygenase-1 (HO-1), and glutathione peroxidase; scavenging ROS; and maintaining intracellular redox homeostasis [24,59]. For example, HO-1 inhibits the release of pro-inflammatory factors by degrading heme to generate antioxidant and anti-inflammatory molecules (e.g., carbon monoxide and biliverdin) [60]. In addition, Nrf2 reduces the inflammatory stimulatory effects of oxidized lipids (e.g., 4-HNE, MDA) on neuronal cells and glial cells by reducing lipid peroxidation [61]. Notably, the Nrf2 and NF-κB signaling pathways do not operate in isolation, and their dynamic antagonism and synergy constitute a central hub for the interactive regulation of oxidative stress and neuroinflammation, further shaping the pathological network of brain aging.

The role of NF-κB in oxidative stress and neuroinflammation is closely linked [62]. Its dual regulatory role in inflammatory response as well as antioxidant defense makes it a target of interest in brain aging exacerbated by environmental pollutants. ROS can activate NF-κB through multiple mechanisms, including the IκB kinase (IKK) pathway, direct oxidative modification, etc. [63,64]. NF-κB induces antioxidant enzymes under moderate oxidative stress, such as Mn-SOD (manganese superoxide dismutase), HO-1 (heme oxygenase-1), Ferritin (ferritin), and other antioxidant genes, thus enhancing cellular antioxidant defenses [65,66], whereas, when oxidative stress is excessive, NF-κB promotes the expression of inflammatory factors and aggravates cellular damage [67].

NLRP3 inflammatory vesicles are important intracellular inflammation-sensing complexes, and their activation is closely related to oxidative stress and mitochondrial damage, which can lead to the release of inflammatory factors (e.g., IL-1β, IL-18). Studies have shown that ROS can directly promote the assembly and activation of NLRP3 [41]. It can also induce protein sulfhydryl modifications (e.g., S-nitroxylation), which affect the function of NLRP3 and its related proteins [68]. And antioxidants (e.g., NAC, Mito-TEMPO) can inhibit NLRP3 activation [69], further demonstrating that ROS play a key role in this process. Mitochondrial damage is also one of the important triggers of NLRP3 inflammatory vesicle activation, which is mainly affected by (1) mitochondrial ROS (mtROS) accumulation [70,71], (2) the release of mitochondrial DNA (mtDNA) [15], and (3) the opening of the mitochondrial membrane permeability transition pore (mPTP) with cytochrome C release [71]. The multiple mechanisms of activation of NLRP3 inflammatory vesicles by mitochondrial damage further highlight the potential value of targeting the oxidative–inflammatory interaction regulatory network in intervening in neurodegenerative processes.

#### 2.4.2. Critical Roles of Other Cell Signaling Pathways in Regulating Oxidative Stress, Neuroinflammation, and Mitochondrial Dysfunction

AMPK is a key regulator of cellular energy homeostasis and plays an important role in mitochondrial function and anti-oxidative stress. It coordinates metabolic reactions by sensing the ATP/AMP ratio and promotes mitochondrial biosynthesis by phosphorylating PGC-1α, while regulating mitochondrial autophagy to maintain its mass [72,73,74,75]. In addition, AMPK reduces ROS generation by decreasing electron leakage and activates the Nrf2 signaling pathway to promote the expression of antioxidant enzymes, which enhances cellular antioxidant defense and mitigates oxidative damage [76,77]. Studies have shown that exposure to PS-MPs leads to ROS accumulation and affects the AMPK-PGC-1α pathway, while the antioxidant NAC reverses this abnormality [78]. In addition, MeHg-induced AMPK activation leads to calcium overload via DRP1-mediated mitochondrial cleavage and MAM formation, and it creates positive feedback with AMPK signaling to promote its neurotoxicity [79]. The synergistic effect of AMPK and Nrf2 reveals a tight coupling between energy metabolism and antioxidant system, while the PI3K/Akt pathway further expands the oxidative–inflammatory–mitochondrial axis regulatory network.

The PI3K/Akt signaling pathway plays an important role in the regulation of oxidative stress, neuroinflammation, and mitochondrial damage. It promotes the expression of antioxidant enzymes (SOD, CAT, GPx) through activation of the Nrf2/ARE pathway, while inhibiting NADPH oxidase activity and reducing ROS generation [80,81]. In neuroinflammation, PI3K/Akt inhibits NF-κB entry into the nucleus by stabilizing IκBα, reduces the expression of pro-inflammatory factors (TNF-α, IL-1β, IL-6), and promotes the secretion of IL-10 to alleviate inflammatory injury [82,83]. In addition, Akt inhibits microglia overactivation and protects neuronal survival [84]. In terms of mitochondrial protection, PI3K/Akt promotes mitochondrial biosynthesis through activation of PGC-1α and inhibits apoptotic signaling to maintain mitochondrial homeostasis [85,86]. It also stabilizes mitochondrial membrane potential, reduces ROS overproduction, and maintains cellular energy metabolism [87]. Studies have shown that both PS-NPs and NiCl_2_ can affect autophagy and apoptosis by down-regulating the PI3K/Akt signaling pathway [88,89]. Overall, the AMPK and PI3K/Akt pathways synergize to maintain the homeostasis of the oxidation–inflammation–mitochondrial axis through the regulation of energy metabolism and antioxidant and anti-inflammatory effects, and their targeted disruption by environmental pollutants reveals a key molecular mechanism in neurodegenerative pathologies.

## 3. Multi-Level Network Construction of Pollutant Small Molecule-Brain Aging

### 3.1. A Multilayer Network Model of the Contaminant Small Molecule Signaling Pathway and Brain Aging

Pollutants affect the brain aging process through multiple pathways, in which aging-related bioactive small molecules act as key mediators to accelerate neuronal degeneration and dysfunction by modulating mechanisms such as oxidative stress, neuroinflammation, and mitochondrial damage. These small molecules are not only direct products or regulators of multiple signaling pathways but also exacerbate the process of brain aging by modulating key signaling pathways (Table 1). In oxidative stress-related pathways, ROS and RNS are the core components of oxidative stress [90]. Low concentrations of ROS regulate antioxidant defense through the Nrf2 pathway, increasing the expression of antioxidant enzymes such as glutathione (GSH) and superoxide dismutase (SOD), thereby resisting oxidative damage [91], whereas at high concentrations, ROS trigger neuroinflammation through the activation of NF-κB and NLRP3 inflammatory vesicles, which promotes the release of cytokines and aggravates neurodegenerative damage [63,92]. Vitamin C helps to maintain oxidative homeostasis by inhibiting the excessive accumulation of ROS and reducing the sustained activation of Nrf2 [93,94]. In addition, vitamin C, as a cofactor of HIF-related hydroxylases (PHDs), can promote HIF-1α degradation and reduce pollutant-induced metabolic abnormalities, thereby inhibiting hypoxia-induced neuroinflammation and energy metabolism disorders [95,96,97]. And vitamin E mainly acts on cell membranes to reduce lipid peroxidation damage and prevent mitochondrial dysfunction [98]. In the neuroinflammatory pathway, prostaglandin E_2_ and arachidonic acid ethanolamine (AEA) play important roles in the regulation of inflammatory responses and synaptic plasticity [99,100]. PGE_2_ promotes the up-regulation of the cAMP/PKA signaling pathway through the activation of the EP2 receptor, and it activates the NF-κB pathway, which further enhances inflammatory responses [99], while AEA regulates nerve excitability and affects NMR via CB1 receptors [101]. AEA regulates neural excitability through the CB1 receptor and affects the calcium overload of the NMDA receptor, which can lead to the accumulation of glutamate in the synaptic gap and excitatory neurotoxicity after exposure to pollutants, resulting in neurological damage [100,102]. Small molecules such as lactate, pyruvate, and ATP regulate cellular energy metabolism and mitochondrial function in the mitochondrial damage pathway, and the AMPK signaling pathway regulates glucose metabolism, fatty acid oxidation, and mitochondrial energy metabolism during hypoxia or metabolic stress to help maintain cellular energetic homeostasis [101,103].

**Figure 6:**
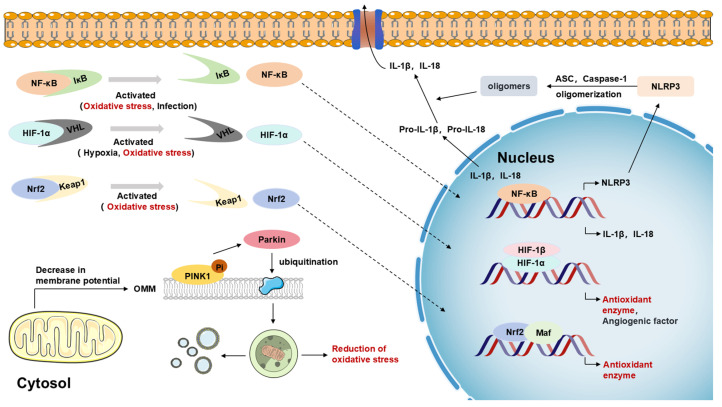
Cascade framework for environmental pollutants exacerbating brain aging.

In the cellular stress response network, the Nrf2, HIF-1α, PI3K/Akt, AMPK, NF-κB, NLRP3, and PINK1/Parkin pathways form a dynamic interaction through the energy–oxidation–inflammation–autophagy axis: AMPK acts as an energy sensor and promotes the initiation of autophagy upon ATP depletion through inhibition of the PI3K/Akt/mTORC1 pathway. AMPK, as an energy sensor, promotes autophagy initiation by inhibiting the PI3K/Akt/mTORC1 pathway upon ATP depletion, and, at the same time, it phosphorylates Nrf2 to enhance its nuclear translocation for activation of antioxidant genes (e.g., HO-1, NQO1) [77,104]. The activation of NF-κB by ROS/TLR4 not only induces the assembly of the NLRP3 inflammasome (through transcriptional upregulation of NLRP3 and pro-IL-1β) but the activation of AMPK also inhibits the NF-κB pathway through the regulation of JNK [105,106]. Also, PINK1/Parkin-mediated mitochondrial autophagy triggered by mitochondrial damage both scavenges ROS sources (in concert with Nrf2) and inhibits NLRP3 activation (Figure 6) [107,108]. These pathways form a sophisticated and fragile regulatory network between redox homeostasis, metabolic reprogramming, and inflammatory abatement. Therefore, it is particularly important to intervene in the signaling pathways of the above small molecules affecting oxidative stress, neuroinflammation, and mitochondrial dysfunction.

### 3.2. Intervention of Exogenous Bioactive Small Molecules

#### 3.2.1. Antioxidant Quantum Dots

The intervention of AQDs in neurological diseases and injuries mainly involves four aspects: oxidative stress, neuroinflammation, mitochondrial damage, and axonal regeneration and remyelination. In terms of oxidative stress, quantum dots can reduce oxidative stress by scavenging free radicals such as OH^−^ and O_2_^−^ [109]; up-regulate antioxidant enzymes, such as SOD, to improve cellular antioxidant capacity [110]; and enhance the Nrf2-mediated antioxidant pathway [111]. In neuroinflammation, quantum dots can inhibit inflammatory signaling pathways such as NLRP3 inflammatory vesicles, NF-κB, and Caspase-1; reduce the release of inflammatory factors [112]; and reduce the toxic aggregation of Aβ to decrease Alzheimer’s disease-related neuroinflammation [113]. In terms of mitochondrial damage, quantum dots can inhibit mitochondrial depolarization and reduce apoptosis [114], reduce mitochondrial iron overload, and restore redox balance [115]. For axonal regeneration and remyelination, quantum dots can promote Schwann cell proliferation and neuroangiogenesis, activate PI3K/Akt and Ras/ERK1/2 signaling pathways, and promote axonal growth and nerve regeneration [116]. Quantum dots can be used for medical imaging and diagnostics due to their fluorescent properties such as high intensity, long lifetime, and photostability. Guo X et al. found that SeQDs can cross the blood–brain barrier (BBB) and be used as a fluorescent probe in AD diagnostic and tracking studies [113]. Huang H et al. found that TPP-Se-CDs have two-photon fluorescence imaging, targeted free radical scavenging in mitochondria, and other functions, which provide a new opportunity for nanomedicine and other functions, providing a new direction for nanomedicine [109].

Although AQDs show great potential in alleviating oxidative stress, neuroinflammation, and mitochondrial damage as well as promoting axonal regeneration and remyelination, and although they can be applied as an imaging and diagnostic tool for the research and treatment of neurological disorders, their further clinical translation is still faced with the challenges of safety and biocompatibility. Therefore, optimizing the biological properties of AQDs and improving their safety for neurological applications have become key directions to promote their practical applications. To further improve the safety and biocompatibility of AQDs in the nervous system, various optimization strategies have been proposed in existing studies. For surface functionalization modification, Sayan et al. successfully enhanced the mechanical properties of the materials and reduced the biotoxicity of carbon quantum dots by surface modification of alginate and cellulose nanofiber hydrogels with carbon quantum dots [117]. For biodegradable material development, Kurungottu et al. synthesized a hybrid composite based on graphene oxide and ZnS (Mn) quantum dots, which significantly improved the biocompatibility of the quantum dots [118]. In terms of delivery optimization, Han et al. successfully delivered quantum dots into the cytoplasm of living human embryonic kidney cells using microelectrophoresis, thereby minimizing damage to the cell membrane [119]. These strategies provide a strong guarantee for the safe application of AQDs in the nervous system.

In recent years, studies have shown that the modification of quantum dots has demonstrated significant advantages over traditional methods across various biomedical applications. Wenxin Qi et al. verified the excellent antimicrobial properties of polyethyleneimine–ethylenediaminetetraacetic acid disodium salt carbon quantum dots in wound treatment, effectively mitigating the risks of bacterial infections and drug resistance commonly linked to traditional pharmacological interventions in clinical settings [120]. Poushali Das et al. developed fluorescent biopolymer composites embedded with silicon-based heteroatom-doped carbon quantum dots, which effectively prevented microbial contamination, thereby enhancing biocompatibility and medical safety [121]. Xinyu Yang et al. designed an amine-functionalized aspirin-derived carbon quantum dot, which exhibited remarkable targeting properties and demonstrated broad clinical application potential [122]. These studies suggest that quantum dot modification in the biomedical field demonstrates potential advantages over traditional methods and holds transformative potential, offering novel strategies to overcome the limitations of conventional therapeutic approaches.

#### 3.2.2. Other Interventions

Exogenous interventions can mitigate the process of brain aging exacerbated by pollutants. In terms of oxidative stress, exogenous small molecule interventions can exert protective effects by modulating antioxidant defense pathways [18,123]. Shu Feng et al. found that the organic small molecule, radical thiol (SFN), had a favorable mitigating effect in oxidative stress caused by methylmercury contamination. SFN pretreatment significantly enhanced the activation of the Nrf2/ARE pathway, which prevented the toxic effects of MeHg exposure [124] in neuroinflammation. Exogenous supplementation of small molecules can be a potential strategy to alleviate neuroinflammation by inhibiting the NF-κB signaling cascade. Jens U Marquardt et al. found that curcumin (CUR), as a potent IKK inhibitor, can directly target and inhibit IKK activity, preventing the phosphorylation and degradation of IκB, thus inhibiting the translocation of NF-κB to the nucleus, and ultimately alleviating the inflammatory response triggered by the inflammatory response triggered by it [125]. Inhibition of the TLR4/NF-κB pathway by curcumin nanoparticles (CNPs) to inhibit microglia polarization has been reported for the treatment of neuroinflammation in Alzheimer’s disease [125]. In terms of mitochondrial stabilization, exogenous supplementation of small molecules to enhance dynamic renewal of intracellular mitochondrial mass is an important way to mitigate the risk of brain senescence. Ying Ann Chiao et al. found that rapamycin enhances the clearance of senescent or damaged mitochondria by transiently activating autophagosome formation. Rapamycin can also upregulate the expression of mitochondrial biosynthesis markers and promote the generation of new mitochondria to replace senescent or damaged mitochondria. This process synergizes with the transient activation of autophagy to achieve dynamic renewal of mitochondrial mass, thereby slowing down aging [126]. Exogenous small molecule supplemental protection of neuronal cells is not only limited to a single pathway, but many small molecules can synchronously activate multiple pathways in vivo to function synergistically. Jinjin Hao and Xuewu Sun et al. found that RTA-408, a good small-molecule inhibitor of the NF-κB pathway, inhibits NF-κB pathway signaling through activation of oxidative stress-related Nrf2 pathway, thereby suppressing neuroinflammatory responses and thus inhibiting neuroinflammatory responses. In addition, RTA-408 can also protect the normal mitochondrial membrane potential in response to oxidative stress and inhibit mitochondrial fission, thus inhibiting apoptosis [127,128,129]. Given the central role of endogenous bioactive small molecules in pollutant-induced brain aging, accurate detection of the levels, distribution, and dynamics of these molecules is essential for an in-depth understanding of the mechanisms of pollutant neurotoxicity. The next section will focus on the current major detection techniques for these bioactive small molecules, including chromatography, mass spectrometry, and molecular fluorescent probes, and discuss their applications in pollutant exposure-related studies.

**Table 1:** Specific pathways by which environmental pollutants affect aging-related bioactive small molecules.

Form	Access	Pollutant	Performance	Specific Impacts	References
oxidative stress	Nrf2	PM_2.5_	increase	The expression of Nrf2 and its downstream antioxidant genes (e.g., NQO1, γ-GCS) is significantly increased	[28]
		lambda-cyhalothrin	increase	Expression of Nrf2 and its downstream genes (e.g., HO-1, NQO1) is upregulated	[29]
		BDE209	increase	Activation of the Nrf2/GPX4 pathway	[10]
		Cd	decrease	Inhibition of the Keap1–Nrf2 pathway and its downstream genes induces lipid peroxidation and ferroptosis	[130]
	HIF-1α	MeHg	decrease	Reduces protein expression levels in astrocytes and inhibits the expression of its multiple downstream target genes	[32]
		Cr^6^⁺	increase	Activates HIF-1α protein and promotes VEGF expression	[34]
		PM_2.5_	increase	Penetration into the lungs and BBB leads to aberrant activation of the HIF-1α signaling pathway, triggering SIRT1/HIF-1α-mediated ferroptosis	[35]
neuroinflammation	NF-κB	PM_2.5_	increase	Increased expression of inflammatory factors and activation of NF-κB pathway	[44]
			increase		
			increase		
		Pb	increase	Inhibits SIRT1 expression and promotes HMGB1 expression, which in turn activates NF-κB	[46]
			increase		
		β-HCH	increase	Regulation of NF-κB and induction of histone acetylation modifications	[45]
			increase		
			increase		
		Fe	increase	Increased expression of α-synuclein in neuronal cells induced by the NF-κB regulatory pathway	[131]
			increase		
	NLRP3	MeHg	increase	NLRP3 inflammatory vesicles are activated by oxidative stress	[11]
		PM_2.5_	increase	NLRP3 inflammatory vesicles are activated by oxidative stress	[28]
		polystyrene	increase	NLRP3 inflammatory vesicles are activated by oxidative stress	[78]
mitochondria	PINK1/Parkin	aluminum chloride	increase	Induces oxidative stress and activates mitochondrial autophagy	[58]
		uBC	increase	Loss of mitochondrial membrane potential and decreased ATP levels	[12]
		PM_2.5_	increase	ROS overproduction and SOD2 expression	[132]
	OXPHOS	MPTP	increase	Inhibits the electron transport chain and exacerbates mitochondrial dysfunction	[57]
		rotenone	increase		
		paraquat	increase		
		PM_2.5_	increase	COX4I1 defects subsequently disrupt OXPHOS, leading to diminished ATP production and ROS accumulation	[133]
			increase	High PM_2.5_ affects mitochondrial OXPHOS and proteins in the electron transport chain	[134]

## 4. Detection of Aging-Related Bioactive Small Molecules Affected by Environmental Pollutants

Our previous studies have demonstrated that environmental pollutants accelerate brain aging through the induction of oxidative stress, neuroinflammation, and mitochondrial dysfunction [135]. Within this pathological cascade, the spatiotemporal dynamics of aging-related bioactive small molecules—including ROS, glutamate, and ATP—serve dual roles as both direct biomarkers of pathological damage and critical signaling hubs regulating cellular pathways [136,137,138]. However, conventional analytical approaches such as chromatography and mass spectrometry remain constrained by limited spatiotemporal resolution and poor in vivo compatibility [139], fundamentally restricting our ability to capture transient fluctuations and network-level interactions within small molecule metabolic systems during pollutant exposure [140]. This technological gap has perpetuated mechanistic overreliance on static, fragmented datasets in the field [141]. Recent advancements in in vivo electrochemical sensing, fluorescence imaging, and microfluidic platforms now provide unprecedented tools for real-time resolution of small molecule dynamics (Table 2) [142,143,144].

### 4.1. Detection of Oxidative Stress-Related Molecules

**Figure 7:**
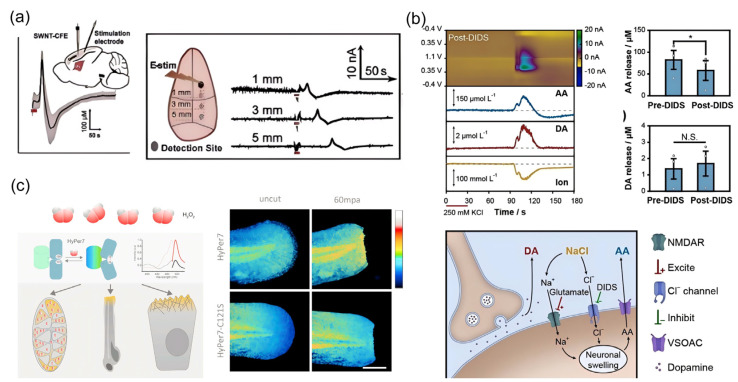
Real-time dynamic detection methods for oxidative stress-related bioactive small molecules. (**a**) In situ electrochemical dynamic detection of vitamin C during spreading depolarization. Reprinted with permission from Ref. [151]. Copyright 2019 Wiley-VCH. (**b**) Data separation based on a deep learning model for simultaneous detection of multiple aging-related bioactive small molecules, Error bars represent the mean square error. Paired t-test, *, p < 0.05; N.S., not statistically significant. Reprinted with permission from Ref. [152]. Copyright 2021 Wiley-VCH. (**c**) Utilization of genetically encoded probes for precise tracking and imaging analysis of ROS. Reprinted with permission from Ref. [150]. Copyright 2020 Elsevier.

Oxidative stress serves as a pivotal mechanism linking environmental pollutant exposure to accelerated brain aging, characterized by the disruption of redox balance through overproduction of ROS/RNS and depletion of endogenous antioxidants [145,146]. Epidemiological evidence consistently suggests that pollutant-induced oxidative damage is associated with neurodegenerative hallmarks, including mitochondrial dysfunction, protein misfolding, and DNA repair defects [147,148]. However, the spatiotemporal dynamics of redox-active molecules—from transient ROS bursts in synaptic clefts to systemic antioxidant exhaustion—remain poorly resolved, primarily due to technical limitations in capturing these transient biochemical events within intact neuronal circuits. The advent of electrochemical detection techniques has made it possible to dynamically monitor changes in redox-active molecules on a temporal scale [149]. Additionally, the introduction of emerging technologies such as genetically encoded biosensors enables real-time tracking of ROS generation and diffusion as well as the precise quantification of antioxidant defense systems [150]. These advancements carry significant implications for measuring brain aging events caused by exposure to environmental pollutants. Building upon pioneering work in electrochemical sensing, Lanquan Mao et al. achieved high spatiotemporal resolution detection of stimulus-evoked vitamin C dynamics in the rat cerebral cortex following spreading depolarization, utilizing SWCNT-modified carbon fiber microelectrodes (Figure 7a) [151]. In subsequent work, this team integrated deep learning architectures with voltammetric detection systems, establishing a multimodal sensing platform capable of simultaneous in vivo monitoring of three aging-related bioactive molecules within intact neural circuits—a critical advancement enabling parallel interrogation of multi-analyte interactions during brain aging (Figure 7b) [152]. Addressing the spatial limitations inherent to conventional electrochemical approaches, which predominantly detect localized redox imbalances, Pak, Valeriy V. et al. engineered the genetically encoded biosensor HyPer7 [150]. This innovation permits high-specificity dynamic monitoring of hydrogen peroxide (H_2_O_2_) diffusion trajectories from mitochondrial matrices to cytosolic compartments, while correlating molecular-scale oxidative events with functional neuroimaging biomarkers (Figure 7c). Collectively, these technological breakthroughs are redefining oxidative stress from a static pathological marker to a spatially organized, dynamically propagating driver of aging-related neural decline.

### 4.2. Detection of Neuroinflammation-Related Molecules

**Figure 8:**
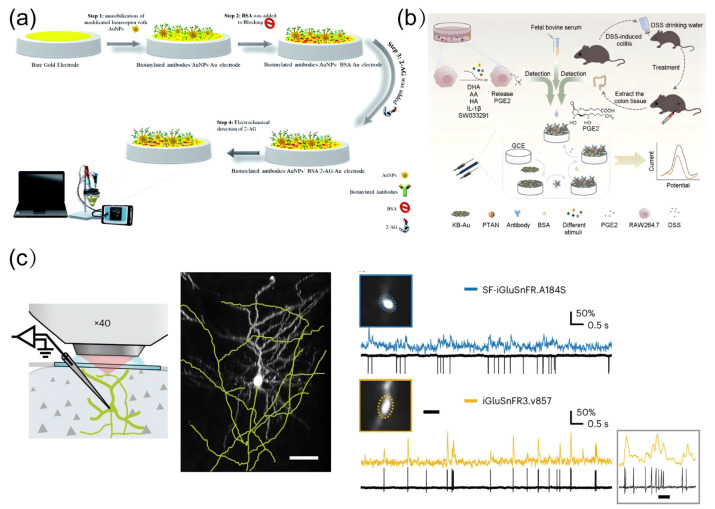
Detection of inflammation-associated bioactive small molecules. (**a**) 2-AG electrochemical immunosensor for detection of inflammatory markers in blood samples. Reprinted with permission from Ref. [162]. Copyright 2022 Royal Society of Chemistry. (**b**) Immunosignal-amplified electrochemical immunosensor for PTAN/Ab. Reprinted with permission from Ref. [163]. Copyright 2024 Elsevier. (**c**) In vivo two-photon glutamate dynamic imaging in the visual cortex. Reprinted with permission from Ref. [164]. Copyright 2024 Springer Nature.

Neuroinflammation, characterized by chronic activation of glial cells and dysregulated release of inflammatory mediators, serves as both a consequence and amplifier of pollutant-driven brain aging [153,154,155]. This self-perpetuating process involves intricate spatial–temporal dynamics of signaling molecules [156,157]. Traditional detection paradigms have largely failed to resolve the rapid, compartmentalized nature of neuroinflammatory cascades [158]. Such limitations obscure critical mechanistic insights, including how transient inflammatory signals transition into sustained neurotoxic microenvironments [159,160]. Emerging multimodal approaches now synergize electrochemical precision, genetically encoded biosensors, and spatial metabolomics to dissect neuroinflammatory networks at unprecedented resolution [161]. Kohansal, Fereshteh et al. pioneered an electrochemical immunosensing platform for 2-arachidonoylglycerol (2-AG) detection by immobilizing 2-AG-specific antibodies onto gold electrodes, enabling sensitive quantification of this endocannabinoid in plasma and serum samples (Figure 8a) [162]. To further enhance the detection limits of electrochemical immunosensors, Zhang, Tingting et al. engineered a signal amplification strategy utilizing poly(thionine-aniline) (PTAN) as a redox mediator, constructing a PTAN/Ab nanocomposite that achieved ultrasensitive detection of prostaglandin E2 released from cellular and tissue matrices (Figure 8b) [163]. Addressing the critical limitation of conventional methods in real-time spatial imaging of targeted biomolecules, Aggarwal, A. et al. designed and optimized iGluSnFR variants with enhanced postsynaptic nanoscale localization (Figure 8c) [164]. This breakthrough enabled in vivo two-photon glutamate imaging within the visual cortex, facilitating real-time visualization of synaptic cleft glutamate accumulation—a hallmark of aberrant neurotransmission during aging.

### 4.3. Detection of Mitochondrial Damage Biomarkers

**Figure 9:**
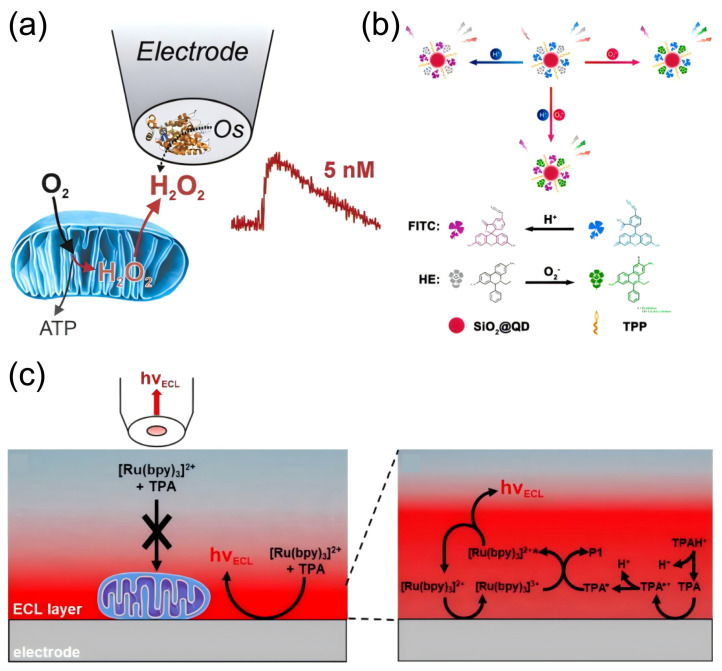
Detection of bioactive small molecules associated with mitochondrial dysfunction. (**a**) Electrochemical monitoring of early events of hydrogen peroxide production by mitochondria. Reprinted with permission from Ref. [169]. Copyright 2014 Wiley-VCH. (**b**) Concurrent identification of bioactive small molecules linked to mitochondrial dysfunction. Reprinted with permission from Ref. [170]. Copyright 2016 American Chemical Society. (**c**) Electrochemiluminescence imaging targeting mitochondria. Reprinted with permission from Ref. [171]. Copyright 2021 Wiley-VCH.

Mitochondrial dysfunction stands as a sentinel event in pollutant-driven brain aging, orchestrating energy crises, redox collapse, and apoptotic cascades through dynamic perturbations in metabolite fluxes and organellar communication [165,166]. Unlike static macromolecular damage, mitochondrial decline manifests as spatiotemporally coordinated failures [167]. Conventional bulk assays, limited to endpoint measurements of isolated mitochondrial fractions, systematically overlook the compartmentalized nature of these pathological processes, failing to resolve how local metabolite imbalances propagate into neuronal network dysfunction [168]. The advent of multimodal real-time monitoring platforms now bridges this critical gap, enabling simultaneous tracking of bioenergetic fluxes, ion gradients, and redox states within functioning mitochondrial networks. These technologies unveil mitochondrial damage not merely as a biomarker of aging but as a dynamic instigator of neurotoxic feedback loops. Suraniti, Emmanuel et al. developed an electrochemical biosensor based on peroxidase-redox polymer-modified electrodes to investigate the central role of mitochondria in cellular redox signaling. This innovation enabled high spatiotemporal resolution monitoring of mitochondria-derived ROS and other aging-related bioactive small molecules during the early stages of mitochondrial dysfunction (Figure 9a) [169]. Building upon this, Huang, Hong et al. engineered a mitochondria-targeted single-fluorescent probe capable of high-precision, selective sensing of pH and superoxide anion (O_2_^−^), thereby facilitating multiplexed detection and real-time imaging of mitochondrial dynamics (Figure 9b) [170]. Leveraging these advancements, Ma, Yumeng et al. achieved electrochemical luminescence imaging of individual mitochondria deposited on electrode surfaces by utilizing classical fluorescent biomarkers and endogenous mitochondrial NADH (Figure 9c). This breakthrough provides a powerful visualization tool for deciphering how environmental pollutants induce mitochondrial dysfunction through small molecule perturbations [171].

**Table 2:** Detection of aging-related bioactive small molecules.

Small Molecule Type	Analyte	Type of Effect	Detection Methods	Innovation Point	Linear Range	Detection Limit	Reference
ROS	H_2_O_2_	Pro-oxidant	Electrochemical Detection	Utilization of the natural mineral amphibole clay as an enzyme immobilization carrier	0.2–150 μM	0.05 ± 0.01 μM	[172]
	O_2_^−^, •OH, and H_2_O_2_			Pt-NWE electrodes	N/A	N/A	[173]
	H_2_O_2_			HRP embedded in crosslinked three-dimensional polymer matrices containing mobile Os redox mediators	1–100 mM	N/A	[169]
RNS	ONOO^−^, NO•, NO_2_^−^	Pro-oxidant	Electrochemical detection	Nanoelectrode	N/A	N/A	[174]
AA	Antioxidant		ECL	Bipolar ratio response	50–3 μM	20 nM	[175]
			Electrochemical detection	SWCNT modified carbon fiber microelectrodes	N/A	N/A	[151]
Glu	Antioxidant		Fluorescent sensors	Superfolded GFP	N/A	N/A	[176]
			Electrochemical detection	Microbial sensors	0–100 mM	1 mM	[177]
Prostaglandin E2	Pro-inflammatory		Electrochemical detection	PTAN-Ab complexes as signal amplification components	10⁻^5^–10^6^ fg/mL	10⁻^5^ fg/mL	[163]
2-AG	Anti-inflammatory		Electrochemical detection	2-AG specific antibody modified electrodes	0.48–1 ngmL^−1^	N/A	[165]
O_2_^−^	Membrane Potential		Fluorescent probe	CdSe/ZnS quantum dots	N/A	N/A	[170]
OXPHOS	Electron transport chain		Real-time cell analysis system	Sphere model	N/A	N/A	[178]
E3 ubiquitin	Autophagic small molecule		Proteomics identification	BioE3	N/A	N/A	[179]
ATP	Membrane Potential		Electrochemical detection	An innovative impedance electrode structure	N/A	N/A	[180]
LAC	Membrane Potential		Enzyme Chromatography, Voltammetry		N/A	N/A	[181]
PA	Membrane Potential		Bacterial luminescence methods	Biospecialty luminescent systems	N/A	N/A	[182]

Note: N/A means not available.

## 5. Conclusions and Outlook

Environmental pollutants accelerate brain aging through multidimensional regulatory networks, with small-molecule metabolic imbalance serving as a key upstream mechanism. Research demonstrates that pollutants induce oxidative stress, neuroinflammation, and mitochondrial dysfunction, disrupting the homeostasis of aging-related small molecules (ROS, PGE2, lactate). This disturbance leads to dysregulation of Nrf2, NF-κB, and AMPK signaling pathways, ultimately triggering cascade effects of macromolecular dysfunction in DNA and proteins. The proposed “metabolic imbalance-signaling dysregulation-macromolecular dysfunction” framework provides new insights into pollutant-induced brain aging. Integrating electrochemical sensing and fluorescence imaging technologies overcomes the spatiotemporal limitations of traditional chromatography/mass spectrometry, enabling precise analysis of metabolic networks and intervention strategies.

Existing methods for detecting brain aging markers rely mainly on imaging and humoral assays, while the detection of bioactive small molecules targeting specific signaling pathways is still at an early stage of exploration. Single-target therapeutic strategies are often ineffective in reversing pollutant-induced brain aging, while combined multi-target interventions may provide superior solutions. Future research could focus on the following directions: developing accurate detection methods for small molecules related to oxidative stress, neuroinflammation, and mitochondrial damage by using high-throughput metabolomics, single-cell sequencing, and biosensing technologies as well as regulating the signaling pathway interaction network to promote the study of multi-targeted joint intervention strategies. Through in-depth analysis of the mechanism of bioactive small molecules in brain aging, it is expected to provide a new scientific basis and application prospects for the prevention and treatment of environmental pollution-related neurodegenerative diseases.

In conclusion, these findings deepen the understanding of pollution-related brain aging mechanisms while providing theoretical and technological foundations for monitoring small molecular indicators of brain aging with high spatiotemporal resolution.

## Data Availability

The data presented in this study are available in this article.

## Abbreviations

DNA	Deoxyribonucleic Acid
ROS	Reactive Oxygen Species
ATP	Adenosine Triphosphate
Nrf2	Nuclear Factor Erythroid 2–Related Factor 2
ARE	Antioxidant Response Element
4-HNE	4-Hydroxynonenal
HIF-1α	Hypoxia-Inducible Factor 1 Alpha
Keap1	Kelch-like ECH-associated protein 1
PM_2.5_	Particulate Matter less than 2.5 μm
Se-Met	Selenomethionine
GPX4	Glutathione Peroxidase 4
VHL	Von Hippel–Lindau
HO-1	Heme Oxygenase 1
NQO1	NAD(P)H Quinone Dehydrogenase 1
LDHA	Lactate Dehydrogenase A
VEGF	Vascular Endothelial Growth Factor
MeHg	Methylmercury
NF-κB	Nuclear Factor Kappa-Light-Chain-Enhancer of Activated B Cells
PI3K	Phosphoinositide 3-Kinase
Akt	Protein Kinase B
FoxO	Forkhead Box O
AMPK	AMP-Activated Protein Kinase
BDE209	Decabromodiphenyl Ether
OS	Oxidative Stress
IκB	Inhibitor of Nuclear Factor Kappa B
TNF-α	Tumor Necrosis Factor Alpha
IL-6	Interleukin 6
NLRP3	NOD-Like Receptor Family Pyrin Domain Containing 3
IL-1β	Interleukin 1 Beta
IL-18	Interleukin 18
IKK	IκB Kinase
κB	Kappa Light Chain Enhancer of Activated B Cells
ASC	Apoptosis-Associated Speck-Like Protein Containing a CARD
BV2	A murine microglial cell line
IL-8	Interleukin 8
β-HCH	Beta-Hexachlorocyclohexane
PINK1	PTEN-Induced Kinase 1
OXPHOS	Oxidative Phosphorylation
SOD2	Superoxide Dismutase 2
GPx	Glutathione Peroxidase
VDAC1	Voltage-Dependent Anion Channel 1
MFN2	Mitofusin 2
SQSTM1	Sequestosome 1
LC3-II	Microtubule-Associated Protein 1A/1B-Light Chain 3-II
ETC	Electron Transport Chain
MPTP	Mitochondrial Membrane Permeability Transition Pore
MPP⁺	1-Methyl-4-Phenylpyridinium
uBC	Ubiquitin C
MDA	Malondialdehyde
Mn-SOD	Manganese Superoxide Dismutase
NAC	N-Acetylcysteine
Mito-TEMPO	Mitochondria-Targeted Tempol
AMP	Adenosine Monophosphate
PGC-1α	Peroxisome Proliferator-Activated Receptor Gamma Coactivator 1 Alpha
PS-MPs	Polystyrene Microplastics
MAMs	Mitochondria-Associated Membranes
CAT	Catalase
RNS	Reactive Nitrogen Species
GSH	Glutathione
AEA	Anandamide
PGE_2_	Prostaglandin E2
EP2	Prostaglandin E Receptor 2
NMR	Nuclear Magnetic Resonance
CB1	Cannabinoid Receptor 1
NMDA	N-Methyl-D-Aspartate
mTORC1	Mechanistic Target of Rapamycin Complex 1
TLR4	Toll-Like Receptor 4
JNK	c-Jun N-terminal Kinase
AQDs	Antioxidant quantum dots
BBB	Blood–brain barrier
SFN	Sulforaphane
CUR	Curcumin
CNPs	Carbon Nanoparticles
RTA-408	A small molecule drug used for neuroprotection
SWCNT	Single-Walled Carbon Nanotube
HyPer7	A genetically encoded hydrogen peroxide sensor
2-AG	2-Arachidonoylglycerol
PTAN	Poly(Thionine-Aniline)
iGluSnFR	A sensor for glutamate in real-time imaging
Pt-NWE	Platinum Nanowire Electrode
ECL	Electrochemiluminescence
GFP	Green Fluorescent Protein
BioE3	A bioactive small molecule used in cellular research
